# Supplementary material for: Disentangling the effects of intermittent faecal shedding and imperfect test sensitivity on the microscopy-based detection of gut parasites in stool samples
Source: PLoS Negl Trop Dis. 2024 Dec 5;18(12):e0012719. doi: 10.1371/journal.pntd.0012719 (PMC11717355; doi:10.1371/journal.pntd.0012719)
Supplement: S1 Table — (PDF) [file pntd.0012719.s002.pdf]

Table S1. The full set of 38 multilevel occupancy models fit to the *Giardia* dataset

Rank	Model	AICc	ΔAICc	w_i	*k*	Deviance
1	Ψ(gender),θ(.),p(observer) [TOP]	485.95	0	0.1189	5	475.73
2	Ψ(gender),θ(age),p(observer)	486.34	0.39	0.0978	6	474.03
3	Ψ(gender+age),θ(.),p(observer)	486.66	0.71	0.0833	6	474.35
4	Ψ(vulnerability+gender),θ(.),p(observer)	486.71	0.76	0.0813	7	472.29
5	Ψ(.),θ(age),p(observer)	487.78	1.83	0.0476	5	477.56
6	Ψ(.),θ(.),p(observer)	487.80	1.85	0.0471	4	479.65
7	Ψ(vulnerability),θ(.),p(observer)	487.80	1.85	0.0471	6	475.49
8	Ψ(age),θ(.),p(observer)	487.86	1.91	0.0457	5	477.64
9	Ψ(.),θ(gender),p(observer)	487.86	1.91	0.0457	5	477.64
10	Ψ(gender),θ(.),p(observer+age)	487.90	1.95	0.0448	6	475.59
11	Ψ(vulnerability+gender),θ(age),p(observer)	488.20	2.25	0.0386	8	471.66
12	Ψ(gender+age_1),θ(age_2),p(observer)	488.45	2.50	0.0341	7	474.03
13	Ψ(vulnerability+gender+age),θ(.),p(observer)	488.49	2.54	0.0334	8	471.95
14	Ψ(gender),θ(.),p(.)	488.88	2.93	0.0275	4	480.73
15	Ψ(vulnerability),θ(age),p(observer)	489.36	3.41	0.0216	7	474.94
16	Ψ(vulnerability+age),θ(.),p(observer)	489.43	3.48	0.0209	7	475.01
17	Ψ(vulnerability+gender+age_1),θ(age_2),p(observer)	489.74	3.79	0.0179	9	471.06
18	Ψ(age_1),θ(age_2),p(observer)	489.86	3.91	0.0168	6	477.55
19	Ψ(gender),θ(order),p(observer)	490.10	4.15	0.0149	7	475.68
20	Ψ(gender),θ(age+order),p(observer)	490.43	4.48	0.0127	8	473.89
21	Ψ(vulnerability+age_1),θ(age_2),p(observer)	490.74	4.79	0.0108	8	474.20
22	Ψ(.),θ(.),p(.) [NULL]	490.75	4.80	0.0108	3	484.66
23	Ψ(vulnerability+gender),θ(order),p(observer)	490.83	4.88	0.0104	9	472.15
24	Ψ(gender+age),θ(order),p(observer)	490.84	4.89	0.0103	8	474.30

25	Ψ(gender),θ(.),p(age)	490.87	4.92	0.0102	5	480.65
26	Ψ(.),θ(age+order),p(observer)	491.82	5.87	0.0063	7	477.40
27	Ψ(vulnerability),θ(order),p(observer)	491.88	5.93	0.0061	8	475.34
28	Ψ(.),θ(order),p(observer)	491.91	5.96	0.0060	6	479.60
29	Ψ(age),θ(order),p(observer)	491.97	6.02	0.0059	7	477.55
30	Ψ(vulnerability+gender),θ(age+order),p(observer)	492.27	6.32	0.0050	10	471.44
31	Ψ(gender+age_1),θ(age_2+order),p(observer)	492.56	6.61	0.0044	9	473.88
32	Ψ(vulnerability+gender+age),θ(order),p(observer)	492.63	6.68	0.0042	10	471.80
33	Ψ(vulnerability),θ(age+order),p(observer)	493.39	7.44	0.0029	9	474.71
34	Ψ(vulnerability+age),θ(order),p(observer)	493.51	7.56	0.0027	9	474.83
35	Ψ(vulnerability+gender+age_1),θ(age_2+order),p(observer)	493.69	7.74	0.0025	11	470.69
36	Ψ(age_1),θ(age_2+order),p(observer)	493.94	7.99	0.0022	8	477.40
37	Ψ(vulnerability+age_1),θ(age_2+order),p(observer)	494.66	8.71	0.0015	10	473.83
38	Ψ(gender),θ(1.0),p(observer) [TOP with θ = 100%]	501.61	15.66	0.0000	5	491.39

AICc, sample size-corrected Akaike's information criterion score; ΔAICc, difference between each model's AICc score and the AICc score of the top-ranking model; w^i, Akaike weight; k, number of estimable parameters; Deviance, –2 log-likelihood
