# Supplementary material for: Disentangling the effects of intermittent faecal shedding and imperfect test sensitivity on the microscopy-based detection of gut parasites in stool samples
Source: PLoS Negl Trop Dis. 2024 Dec 5;18(12):e0012719. doi: 10.1371/journal.pntd.0012719 (PMC11717355; doi:10.1371/journal.pntd.0012719)
Supplement: Attachment — Submitted filename: Ferreira-Sa et al. Overall Comments.docx [file pntd.0012719.s005.docx]

**Overall Comments**

**Authors:** Lana C.E. Ferreira-Sá, Eleuza R. Machado, Rodrigo Gurgel-Gonçalves, Fernando Abad- Franch

**Title:** Disentangling the effects of intermittent faecal shedding and imperfect test

sensitivity on the microscopy-based detection of gut parasites in stool samples

**Review Questions**

**Key Review Criteria Required for Acceptance?**

As you describe the new analyses required for acceptance, please consider the following:

**Methods**

-Are the objectives of the study clearly articulated with a clear testable hypothesis stated?
-Is the study design appropriate to address the stated objectives?
-Is the population clearly described and appropriate for the hypothesis being tested?
-Is the sample size sufficient to ensure adequate power to address the hypothesis being tested?
-Were correct statistical analysis used to support conclusions?
-Are there concerns about ethical or regulatory requirements being met?

Response: The authors apply a hierarchical modeling approach to estimate *Giardia* infection in children attending kindergartens in the Federal District, Brazil while accounting (estimating) for stool-sample–level shedding rates and imperfect test-level detection. This type of approach is appropriate, but rarely applied, to disentangling the effects of intermittent shedding and imperfect sensitivity and better understand transmission dynamics, disease epidemiology, and diagnostic-test performance. We applaud the authors for applying such an approach and provide several suggestions to make the use of this approach more appealing to the journal’s audience and disease ecology in general. Below, we highlight several general suggestions, but also include smaller, editorial suggestions in the attached pdf.

1. The authors may consider enhancing the biological justification for their hypotheses (i.e., models). This could include a little more background on *Giardia* – it is a gut parasite, but how is it likely transmitted among individuals (i.e., children) in this system. The authors give some background on lines 113-127, but it is not clear how differentiating intermittent parasite shedding and imperfect test sensitivity helps manage, contain, or control pediatric *Giardia* infections. How does this enhanced understanding lead to better treatment for such diseases in this community, or similar communities?
2. This understanding is particularly important to appreciate some of the models, and model covariates, investigated in this study. In theory, the covariates (lines 209-229) relate to hypotheses the authors want to test with their data but understanding the reasoning for some of these hypotheses (covariates) is not clear. For example:
   a. The authors state that children were sampled from kindergartens in four urban sectors of the Federal District, Brazil with different degrees of overall ‘social vulnerability’. What criteria were used to determine ‘social vulnerability’? What makes children in one kindergarten more ‘socially vulnerable’ to pediatric *Giardia* infections than other communities/kindergartens?
   b. The authors state that the probability that a child has *Giardia* infections may be higher with older children (age = months) or that older children may have higher intermittent shedding rates (probabilities), given infection (θ), because Giardia gut-population densities tended to increase with time since infection. The authors state that age is standardized (mean 0, sd = 1) but its not clear that they treated age as a continuous covariate. Table 2 suggests that the age were grouped into two categories: children 4-36 months; children 37-73 months. What is the significance of this grouping with respect to a child having *Giardia* infections or shedding environment-resistant infectious cysts in their stool? If the authors used age as a continuous variable, then I would suggest not giving data in age categories (e.g. Table 2) and reporting and graphing the relationship between a given parameter (e.g., theta, ϑ) and age over the range of values in their data (4-73 months), using a supported model (Model 2: ψ(gender) ϑ(age) p(observer); model weight =0.39). Similarly, the authors could have reported the slight effect of age on a child having *Giardia* infections (ψ), using a supported model (Model 3: ψ(gender+age) ϑ(.) p(observer); model weight =0.71).
   c. The authors could provide some justification for why they considered time-ordered serial samples as a covariate if they did not expect this to influence shedding probability.
3. Tables 1 and 2 seem a little redundant to me. Is Table 1 necessary? Alternatively, it might be possible to remove Table 2 or move to supplemental information since some of the highlights are given in this paragraph. Another option would be to retain Table 2 and add model-based estimates for these same levels.
4. Data Analysis Section:
   a. We are not sure explaining the multilevel occupancy models in terms of its historic use (i.e., with patches of habitat) is that useful to this audience. It might be a distraction. Instead, the authors could use the term 'units' instead of 'sites'. MacKenzie et al. 2017 chose to do so because of work like this that points out multi-level occupancy can be applied to units other than habitat patches. If the authors adopt this terminology, then the sentence on lines 248-250 would read:

“Multilevel site-occupancy models consider a hierarchy of (1) units (e.g., individuals), which may be occupied (with probability Ψ) by the target parasite/pathogen species; (2) sub-units or occasions, in/during which the target species, if present at the unit, may be available for detection, and...”

Additional changes may be needed later, if the authors adopt this terminology.

b. In addition to the comments regarding the authors hypotheses and associated covariates, the authors should provide more detail regarding their **model building and model selection** approach. Specifically:
   i. Provide more detail on ‘the series of exploratory analyses to preliminarily assess the

evidence, if any, for (i) intermittent Giardia shedding, (ii) imperfect test sensitivity, and (iii) variation in observed child-level infection, sample-level shedding, and test-level detection’. How did this lead to your candidate model set?

ii. I would like to see a bit more description in the supporting material for the model building and model selection process. For example, there is something called age_1 and age_2, which appears different from 'age'.
iii. Some of the models that they mention in the text (e.g., ψ(vulnerability) ϑ(.) p(.)) are not part of this model set.
iv. The authors might consult Morin et al. 2020 for a model building strategy:

Morin, D.J., Yackulic, C.B., Diffendorfer, J.E., Lesmeister, D.B., Nielsen, C.K., Reid, J. and Schauber, E.M., 2020. Is your ad hoc model selection strategy affecting your multimodel inference?. Ecosphere, 11(1), p.e02997.

Their candidate model set is not balanced, and I question if that influences some of their inference is simply because of the lack of balance. For example, it’s not surprising that there was no effect of age on detection probability if only one model contained that structure, and the remaining 37 models did not. Similarly, it’s not surprising that there was no effect of gender on shedding probability if only one model contained that structure, and the remaining 37 models did not. Again, I would refer the authors to Morin et al. 2020 for more defendable, step-wise approach to model building and model selection.

**Results**

-Does the analysis presented match the analysis plan?
-Are the results clearly and completely presented?
-Are the figures (Tables, Images) of sufficient quality for clarity?

(Limit 20000 Characters)

Response: The writing and communication of the results could be improved – see specific comments in the attached document. I do not think it is necessary to repeat the information included in the model selection Table 3 (Lines 338-354); instead, the authors should provide a better biological interpretation of these results. First, I would verify that your model selection table is balanced - i.e., verify that gender, age, and vulnerability appear in the same number of child-level Giardia infection (Ψ) structures. Similarly, verify that age, gender, order, and null (.) structures appear in the same number of stool-level Giardia infection (ϑ) structures. Again, see Morin et al. 2020 on various step-wise model building strategies that insure a balanced model set.

In this candidate set, model 6 Ψ(.),θ(.),p(observer) is really your ‘null’ model for evaluating factors influencing child-level Giardia infection probability (Ψ) and stool-level Giardia infection or shedding probability (ϑ). If I were interpreting these results, I would say there is strong evidence that Giardia infection probability (Ψ) varies with gender and is higher for girls (Ψ Girls = 0.54; SE(Ψ Girls) = 0.14) compared to boys (Ψ boys = 0.34; SE(Ψ boys) = 0.10; estimates based on the best-supported model, Figure 3). The gender effect is included in each of the top 4 models and 4 of the 5 models that perform better than the null model (Model 6, S1 Table). There is some evidence for an additive effect of age ($\hat{\beta}_{age}=0.26$; SE($\hat{\beta}_{age})$= 0.23) and social vulnerability ($\hat{\beta}_{int}=-1.00$; SE($\hat{\beta}_{int})$= 0.87; $\hat{\beta}_{high}=-0.20$; SE($\hat{\beta}_{high})$= 0.97) based on the best-supported model with these covariates, but the effects are imprecise. We do note that the direction of the effects is consistent with our expectations, with higher probabilities of Giardia infection probabilities for older children and those that reside in the Intermediate and High vulnerability sectors of the Federal District. The best-supported structure (hypothesis) for shredding probability suggests that this probability does not vary among sample order, gender, or age. The probability of stool-sample–level Giardia shedding, given child infection was ($\hat{\theta}=0.44$; SE($\hat{\theta}$=0.12); estimates based on the best-supported model, S1 Table). There was some evidence that the shedding probability was slightly higher for older children ($\hat{\beta}_{theta-age}=0.31$; SE($\hat{\beta}_{theta-age})$= 0.25) based on the second-best model, but these estimates are imprecise. This paragraph illustrates how you can use the model estimates together with the model selection table to interpret the result for your reader.

I know there is some model uncertainty in your candidate model set, but I don’t think the model-averaged estimates are that useful or necessary. I think you could include some of those estimates as an added column in Table 2 (if you chose to retain it) to illustrate the bias that exists using the raw proportions, but I don’t think Figure 4 is that useful or informative. A figure showing the relationship between child age (continuous variable) and shedding probability, based on the 2^nd^ best-supported model would probably be more interesting.

**Conclusions**

-Are the conclusions supported by the data presented?
-Are the limitations of analysis clearly described?
-Do the authors discuss how these data can be helpful to advance our understanding of the topic under study?
-Is public health relevance addressed?

Response: In general, I believe the conclusions supported in the Discussion section reflect the data analysis well. I only have minor editorial comments on this section (see attached pdf).
