# Supplementary material for: Disentangling the effects of intermittent faecal shedding and imperfect test sensitivity on the microscopy-based detection of gut parasites in stool samples
Source: PLoS Negl Trop Dis. 2024 Dec 5;18(12):e0012719. doi: 10.1371/journal.pntd.0012719 (PMC11717355; doi:10.1371/journal.pntd.0012719)
Supplement: Attachment — Submitted filename: Giardia PNTDs Responses to Reviewers.docx [file pntd.0012719.s006.docx]

Dear editor,

We first want to thank the Reviewers for their thorough assessment of our submission. Below we provide (in red font) point-by-point responses to each of the points they raised. We have uploaded a revised version (R1) with changes highlighted in yellow and a ‘clean’ version (uploaded as the manuscript file). We hope that this revised version meets the standards of PLOS NTDs, and look forward to hearing from you.

Sincerely,

Rodrigo Gurgel-Gonçalves (on behalf of all co-authors).

P.S. – We have changed the names of our supplementary files to match those in the main text.

**Methods**

-Are the objectives of the study clearly articulated with a clear testable hypothesis stated?

-Is the study design appropriate to address the stated objectives?

-Is the population clearly described and appropriate for the hypothesis being tested?

-Is the sample size sufficient to ensure adequate power to address the hypothesis being tested?

-Were correct statistical analysis used to support conclusions?

-Are there concerns about ethical or regulatory requirements being met?

Reviewer #1: yes

Thanks.

Reviewer #2: The methods are well described.  The authors take a very technical approach to answering a very technical questions.  Nevertheless I agree with the authors that their manuscript will be of interest to the NTD community, and is applicable to a lot of NTDs.  I am afraid that I no familiar with the multilevel ecological modeling approach they've taken, and cannot confidently comment on its appropriateness.  However it seems sounds to my uneducated understanding.

We thank the Reviewer for these positive comments.

I may have missed this, but it would be good to understand how the authors dealt with missing samples. It look they had 276 children over three week, which is 828 samples.  However, there were only 413 samples in the results.  How was missinginess accounted for, and how might affect the results?  It is very understandable that sample collection approach (this age group, and done by parents) would result in missing samples, but it would just be good to think about how this affects the results.

In our approach, missing observations are simply skipped when computing the likelihood (see, e.g., [12]). For example, the probability of observing each of the following two ‘detection histories’ (in which “1” = detection, “0” = non-detection, and “-” = missing observation) is:

History A: **00 10 00** (sample 1: none of two tests detected Giardia [“**00**”]; sample 2: one test detected *Giardia*, the other one did not [“**10**”]; sample 3: no test detected Giardia [“**00**”])

Pr(History A) = Ψ x [θ x (1-p) x (1-p) + (1-θ)] x [θ x (p) x (1-p)] x [θ x (1-p) x (1-p) + (1-θ)]

History B: **00 10 --** (sample 1: no test detected Giardia [“**00**”]; sample 2: one test detected *Giardia*, the other one did not [“**10**”]; sample 3: not collected, so that the observations are missing [“**--**”])

Pr(History B) = Ψ x [θ x (1-p) x (1-p) + (1-θ)] x [θ x (p) x (1-p)]

Note that the difference between these two equations is that the part highlighted in blue in the first equation (i.e., the part describing observations stemming from the tests run on the third sample) is skipped in the second equation.

The main adverse effect of such missing observations is that we have a smaller amount of information than we would if the data were complete – and that fact, ultimately, leads to more uncertainty and yields larger standard errors (see, e.g., lines 512-518 – to which we have now added that “…our models accommodate missing observations […] by skipping them in the computation of the likelihood…”).

Reviewer #3: The authors apply a hierarchical modeling approach to estimate Giardia infection in children attending kindergartens in the Federal District, Brazil while accounting (estimating) for stool-sample–level shedding rates and imperfect test-level detection. This type of approach is appropriate, but rarely applied, to disentangling the effects of intermittent shedding and imperfect sensitivity and better understand transmission dynamics, disease epidemiology, and diagnostic-test performance. We applaud the authors for applying such an approach and provide several suggestions to make the use of this approach more appealing to the journal’s audience and disease ecology in general. Below, we highlight several general suggestions, but also include smaller, editorial suggestions in the attached pdf.

Thanks for the encouraging remarks and useful suggestions.

1. The authors may consider enhancing the biological justification for their hypotheses (i.e., models). This could include a little more background on Giardia – it is a gut parasite, but how is it likely transmitted among individuals (i.e., children) in this system. The authors give some background on lines 113-127, but it is not clear how differentiating intermittent parasite shedding and imperfect test sensitivity helps manage, contain, or control pediatric Giardia infections. How does this enhanced understanding lead to better treatment for such diseases in this community, or similar communities?

To further clarify that *Giardia* transmission is through the faecal-oral route, we have added some detail to the sentence in l. 116-118, which now reads: “*Giardia* is a major global cause of diarrhoea, and transmission involves faecal shedding of environment-resistant infectious cysts that may be then be ingested by a susceptible host…”.

In lines 108-111 we provide what we think is a brief answer to the Reviewer’s closing question – namely, that “…knowing $\theta$ would provide crucial insight into parasite shedding, which is a key driver of transmission, and knowing $p$ would provide crucial insight into test performance, which is key to interpreting the outcome of diagnostic procedures and to estimating epidemiological parameters including prevalence, incidence, or cure rates.” More generally, we open our Discussion with a paragraph explaining why shedding is key to pathogen transmission (also in our Fig 5), and why separately estimating shedding and narrow-sense test sensitivity is “…critical to understand pathogen transmission and infection diagnosis…” (l. 429-430).

From a more practical point of view, we note that the third paragraph of the Discussion is devoted to answer the explicitly formulated question, “How can clinical sensitivity be enhanced?” (l. 472). We then suggest and illustrate several strategies that would lead to enhanced diagnostic accuracy (and, therefore, better chances of adequate case management) in real-world settings (l. 472-492).

In the fourth paragraph of the Discussion (l. 493-508), we turn to the question of how “[f]ormal estimates of shedding probabilities can […] provide insight into pathogen transmission dynamics” (l. 493-495); we stress that “…the ability to separately estimate and model $\theta$ opens the possibility of asking crucial questions about the underpinnings of among-host variance in infectivity…”, and then focus on the little-understood fact that, “…for many pathogens, only a few individual hosts are ‘super-spreaders’ that account for most transmission events…” (l. 496-499).

2. This understanding is particularly important to appreciate some of the models, and model covariates, investigated in this study. In theory, the covariates (lines 209-229) relate to hypotheses the authors want to test with their data but understanding the reasoning for some of these hypotheses (covariates) is not clear. For example:

a. The authors state that children were sampled from kindergartens in four urban sectors of the Federal District, Brazil with different degrees of overall ‘social vulnerability’. What criteria were used to determine ‘social vulnerability’? What makes children in one kindergarten more ‘socially vulnerable’ to pediatric Giardia infections than other communities/kindergartens?

We first wish to note that our primary aim was to *illustrate* how Ψ, θ, and *p* can be estimated separately in a gut parasite-human host system; estimating covariate effects on those parameters was more of a ‘secondary’ goal (see, e.g., l. 112-114, 220-222, 524-528).

Contextual ‘social vulnerability’ was taken from ref. [21]. We have now included a brief explanation (l. 140-142: “Social vulnerability is measured by 19 sector-level indicators of urban infrastructure and environment; human capital and education; and income and employment [21]”). We stress, here and in the manuscript, that this is *overall* or *contextual*, *city sector-level* vulnerability (e.g., l. 139, 140-142, 223-224, 311, 355, 402…).

b. The authors state that the probability that a child has Giardia infections may be higher with older children (age = months) or that older children may have higher intermittent shedding rates (probabilities), given infection (θ), because Giardia gut-population densities tended to increase with time since infection. The authors state that age is standardized (mean 0, sd = 1) but its not clear that they treated age as a continuous covariate. Table 2 suggests that the age were grouped into two categories: children 4-36 months; children 37-73 months. What is the significance of this grouping with respect to a child having Giardia infections or shedding environment-resistant infectious cysts in their stool? If the authors used age as a continuous variable, then I would suggest not giving data in age categories (e.g. Table 2) and reporting and graphing the relationship between a given parameter (e.g., theta, ϑ) and age over the range of values in their data (4-73 months), using a supported model (Model 2: ψ(gender) ϑ(age) p(observer); model weight =0.39). Similarly, the authors could have reported the slight effect of age on a child having Giardia infections (ψ), using a supported model (Model 3: ψ(gender+age) ϑ(.) p(observer); model weight =0.71).

Please note that those “statements” are ‘just’ hypotheses, which we code into models to illustrate how our approach can help us better understand pathogen biology and disease epidemiology. Age (in months) was modeled as a continuous covariate; we have clarified this in l. 225-226: “covariate ‘age’; months, continuous, standardised to mean 0 and SD 1”. We provide descriptive results in two age classes just to show that infection might have been slightly more common among older children (21 vs 19%), stool samples from older positive children might have had more chances of containing cysts (72 vs 66%), and tests did not seem to detect cysts more often in positive samples from older or younger children (67 and 70%, respectively). We have added a clarification to the footnote of Table 2 (l. 311-312: “…(ii) child age in months (with children grouped, for illustrative purposes, in two strata—younger *vs*. older than approximately 3 years)…”).

Please note that “0.39” and “0.71” are not the ‘weights’ of Models 2 and 3; those figures are ΔAICc values, i.e., the differences between each model’s AICc and the top model’s AICc. Model weights were 0.1 for Model 2 and 0.08 for Model 3 (column *w*_i_ in Table 3).

c. The authors could provide some justification for why they considered time-ordered serial samples as a covariate if they did not expect this to influence shedding probability.

Just because, as noted above, we aimed at illustrating how our approach can be used to test hypotheses — even if those hypotheses are not very plausible a priori. (As an aside, that is exactly what most analysts do when running null-hypothesis tests — test highly implausible null hypotheses that the analysts usually don’t believe to be true and almost never *want* to be true.)

3. Tables 1 and 2 seem a little redundant to me. Is Table 1 necessary? Alternatively, it might be possible to remove Table 2 or move to supplemental information since some of the highlights are given in this paragraph. Another option would be to retain Table 2 and add model-based estimates for these same levels.

We see the point of the Reviewer, but believe that Table 1 provides an overview of the study population that Table 2 does not. (But, if the Editors find that Table 1 would better appear as a Supplementary Table, we are ready to do this.)

4. Data Analysis Section:

a. We are not sure explaining the multilevel occupancy models in terms of its historic use (i.e., with patches of habitat) is that useful to this audience. It might be a distraction. Instead, the authors could use the term 'units' instead of 'sites'. MacKenzie et al. 2017 chose to do so because of work like this that points out multi-level occupancy can be applied to units other than habitat patches. If the authors adopt this terminology, then the sentence on lines 248-250 would read:

“Multilevel site-occupancy models consider a hierarchy of (1) units (e.g., individuals), which may be occupied (with probability Ψ) by the target parasite/pathogen species; (2) sub-units or occasions, in/during which the target species, if present at the unit, may be available for detection, and...”

Additional changes may be needed later, if the authors adopt this terminology.

We now use ‘spatial units’, thanks (see l. 259-264).

b. In addition to the comments regarding the authors hypotheses and associated covariates, the authors should provide more detail regarding their model building and model selection approach. Specifically:

i. Provide more detail on ‘the series of exploratory analyses to preliminarily assess the evidence, if any, for (i) intermittent Giardia shedding, (ii) imperfect test sensitivity, and (iii) variation in observed child-level infection, sample-level shedding, and test-level detection’. How did this lead to your candidate model set?

Our hypotheses (and the models that represent them) were formulated before seeing the data. We then tried to derive predictions from those a priori hypotheses. In this case, as we write in l. 244-247, “…[t]he hypothesis of intermittent faecal shedding predicts that not all samples drawn from children known to be infected will contain *Giardia*, and the hypothesis of imperfect test sensitivity predicts that not all tests run on samples known to contain *Giardia* will yield *Giardia* detections…”. We then looked at what the data said about those predictions, and give the results in Table 2.

ii. I would like to see a bit more description in the supporting material for the model building and model selection process. For example, there is something called age_1 and age_2, which appears different from 'age'.

Those two ‘ages’ just indicate that in some models, child age (which is one and the same for each child) can affect the probabilities of *both* infection (‘age_1’) and shedding (‘age_2’). We agree that this is confusing and have removed the “_1” and “_2” from S1 Table.

iii. Some of the models that they mention in the text (e.g., ψ(vulnerability) ϑ(.) p(.)) are not part of this model set.

Thanks for spotting this; we now use a model in the set (with ‘gender’ instead of ‘vulnerability’) to illustrate the link between hypotheses and models (l. 271-273).

iv. The authors might consult Morin et al. 2020 for a model building strategy:

Morin, D.J., Yackulic, C.B., Diffendorfer, J.E., Lesmeister, D.B., Nielsen, C.K., Reid, J. and Schauber, E.M., 2020. Is your ad hoc model selection strategy affecting your multimodel inference? Ecosphere, 11(1), p.e02997.

Their candidate model set is not balanced, and I question if that influences some of their inference is simply because of the lack of balance. For example, it’s not surprising that there was no effect of age on detection probability if only one model contained that structure, and the remaining 37 models did not. Similarly, it’s not surprising that there was no effect of gender on shedding probability if only one model contained that structure, and the remaining 37 models did not. Again, I would refer the authors to Morin et al. 2020 for more defendable, step-wise approach to model building and model selection.

The model set does not need to be ‘balanced’ for model-averaged estimates to be reliable; ‘imbalance’ would be a problem if we aimed at providing a quantification of “covariate importance” (see ref. [36]), but that was not the case in our analyses. Please note that our model set was specified a priori (except for the “variations of the top-performing model” described in l. 363-365), so that the considerations about ‘multi-stage model selection strategies’ discussed by Morin et al. do not directly apply to our analyses. As Morin et al., in any case, put it (p. 14), “[m]ultimodel inference was […] intended […] to make inference about a priori hypotheses *conditional on the proposed candidate set* (Burnham and Anderson 2002)”, where “Burnham and Anderson 2002” is our ref. [36] and the emphasis is ours. They then go on to say that analysts “…must first make hard decisions about the specific hypotheses they care to evaluate…”, again citing our ref. [36]. That describes well our approach to modeling.

Reviewer #4: This section is well-detailed and explains the processes sufficiently to ensure reproducibility. It's also fascinating to see how a model originally used in wildlife ecological studies is being applied to medical research.

We thank the Reviewer for these encouraging remarks.

However, two points stand out:

While the primary focus is elsewhere, the sampling process in the kindergartens could be described in more detail. For example, were all children in the kindergartens included, or just a subset? How was the sampling conducted, and was there any calculation for sample size? Were there children who opted out of the study? Were any of the children under treatment for conditions such as diarrhea?

There was no calculation for sample size; instead, all the children in each kindergarten were invited to participate in the study, and all those whose guardians provided informed consent and stool samples were included in our analyses (clarified in l. 142-144). No child was under treatment for gut parasites or potentially associated conditions such as diarrhoea during or shortly before the sampling period (clarified in l. 147-148).

While it makes theoretical sense to exclude ambiguous reports to assume 100% specificity, it is unclear whether these ambiguous results were more common in the junior observer’s reports. If this were the case, excluding them could potentially skew the observer covariate calculations. Reporting the number (or at least the approximate percentage) of excluded cases could also be informative.

Our study design, including sampling and testing (lab) protocols, reflected the methodological aim of using multilevel site-occupancy models; ambiguous results were therefore not recorded.

**Results**

Reviewer #1: yes

Reviewer #2: Yes, the results are good.  Again, I am not an expert in this modeling approach, but it is relatively well presented.

Thanks.

Reviewer #3: The writing and communication of the results could be improved – see specific comments in the attached document. I do not think it is necessary to repeat the information included in the model selection Table 3 (Lines 338-354); instead, the authors should provide a better biological interpretation of these results. First, I would verify that your model selection table is balanced - i.e., verify that gender, age, and vulnerability appear in the same number of child-level Giardia infection (Ψ) structures. Similarly, verify that age, gender, order, and null (.) structures appear in the same number of stool-level Giardia infection (ϑ) structures. Again, see Morin et al. 2020 on various step-wise model building strategies that insure a balanced model set.

Please see our response above on ‘balanced’ model sets and multi-stage or step-wise model building. In essence, (i) since we did not aim at quantifying “covariate importance”, covariate ‘imbalance’ does not affect our results, and (ii) since our model set was specified a priori, considerations about ‘multi-stage model selection strategies’ do not directly apply to our analyses.

In this candidate set, model 6 Ψ(.),θ(.),p(observer) is really your ‘null’ model for evaluating factors influencing child-level Giardia infection probability (Ψ) and stool-level Giardia infection or shedding probability (ϑ). If I were interpreting these results, I would say there is strong evidence that Giardia infection probability (Ψ) varies with gender and is higher for girls (Ψ Girls = 0.54; SE(Ψ Girls) = 0.14) compared to boys (Ψ boys = 0.34; SE(Ψ boys) = 0.10; estimates based on the best-supported model, Figure 3). The gender effect is included in each of the top 4 models and 4 of the 5 models that perform better than the null model (Model 6, S1 Table). There is some evidence for an additive effect of age (β ̂_age=0.26; SE(β ̂_age)= 0.23) and social vulnerability (β ̂_int=-1.00; SE(β ̂_int)= 0.87; β ̂_high=-0.20; SE(β ̂_high)= 0.97) based on the best-supported model with these covariates, but the effects are imprecise. We do note that the direction of the effects is consistent with our expectations, with higher probabilities of Giardia infection probabilities for older children and those that reside in the Intermediate and High vulnerability sectors of the Federal District. The best-supported structure (hypothesis) for shredding probability suggests that this probability does not vary among sample order, gender, or age. The probability of stool-sample–level Giardia shedding, given child infection was (θ ̂=0.44; SE(θ ̂=0.12); estimates based on the best-supported model, S1 Table). There was some evidence that the shedding probability was slightly higher for older children (β ̂_(theta-age)=0.31; SE(β ̂_(theta-age))= 0.25) based on the second-best model, but these estimates are imprecise. This paragraph illustrates how you can use the model estimates together with the model selection table to interpret the result for your reader.

We thank the Reviewer for their suggestions, but feel that our focus on probabilities (rather than betas, which we provide in S1 Text) is more intuitive.

I know there is some model uncertainty in your candidate model set, but I don’t think the model-averaged estimates are that useful or necessary. I think you could include some of those estimates as an added column in Table 2 (if you chose to retain it) to illustrate the bias that exists using the raw proportions, but I don’t think Figure 4 is that useful or informative. A figure showing the relationship between child age (continuous variable) and shedding probability, based on the 2nd best-supported model would probably be more interesting.

Again, we appreciate the Reviewer’s suggestions, but feel (i) that our illustration of the approach is made more complete by also showing that it can be used to easily get model-averaged estimates (with unconditional SEs), and (ii) that Fig 4 helps illustrate the richness of information (including information on the uncertainty around predicted probabilities) that one may derive from these models.

Reviewer #4: This section, along with the tables and figures, is appropriate, precise, and of high quality, aiding in understanding the findings.

I would like to ask the authors why statistical tests to assess the significance of differences (such as P values) were not applied in Table 2. Was this outside the scope of the study, or were there specific technical considerations?

In line with a vast and growing body of literature on the downsides of null-hypothesis testing (especially in observational data analysis), we decided not to include formal tests of the null (and p-values) in our exploratory analyses. We prefer to focus instead on the much easier to interpret, and intuitive, probability-scale observations (as in Table 2) and model predictions (as in Figs 3 and 4).

**Conclusions**

Reviewer #1: yes

Reviewer #2: The conclusion are very reasonable.  I like that authors have highlighted that certain molecular techniques might get around the issue of intermittent shedding.

Thanks.

Reviewer #3: In general, I believe the conclusions supported in the Discussion section reflect the data analysis well. I only have minor editorial comments on this section (see attached pdf).

Thank you, we have gone through and accepted most of the suggestions.

Reviewer #4: As suggested in the journal guidelines, I prefer that the discussion begin with a summary of the key findings. Currently, the first paragraph of the discussion reads more like introductory material.

Because we want to emphasize the methodological aspects of our work, rather than the specific findings about our case-study (on *Giardia*), we would prefer to keep our phrasing.

The results of the models in this paper are striking, if not shocking. For example, the estimated infection rate is almost double the observed infection rate. Likewise, the estimated infection rate for girls is approximately 1.6 times higher than for boys. Moreover, 70-80% of Giardia infections may go undetected with a one-sample/one-test strategy. Are these large figures reliable, or are there doubts about them? Is there any evidence from other populations to support these numbers? Is there a way to validate these estimates? Additionally, do existing studies corroborate the estimations, like gender differences in Giardia infection? These findings are significant enough to warrant further discussion on their reliability.

We believe our results are reliable, but would expect the values to differ in other settings — i.e., other populations, tests, observers, etc. We found a few studies in which two-level hierarchical models were used to estimate the ‘clinical sensitivity’ (i.e., θ x *p*) of parasitological tests for *Giardia*. They reported values ranging from ~0.14 to ~0.58 [28,41] — i.e., values roughly compatible with our finding that clinical sensitivity may be alarmingly low for *Giardia* when using a one-sample-one-test strategy. We now mention these results, but explicitly call for caution in comparing numerical study outcomes (l. 454-459).

It might also be worthwhile for the authors to elaborate on how these findings contribute to the current body of knowledge. The limitations are well-explained and realistic. As suggested by the journal, I would recommend that the authors also provide suggestions for future research in this section.

Our Title and last paragraph are examples of our attempts at summarizing the overall relevance of this contribution – i.e., that we show how to disentangle θ from *p*. Instead of pinpointing specific research directions, we prefer to emphasize that we present “…an approach that may find application in a wide variety of pathogen–host systems…” (l. 527-528), as illustrated also by our Fig 5 and, e.g., lines 42-46, 61-63, or 127-130.

**Editorial and Data Presentation Modifications**

Reviewer #1: accept

Reviewer #2: (No Response)

Reviewer #3: See attached pdf with minor recommendations.

Reviewer #4: The supplementary information is comprehensive and helpful, and I appreciate the authors’ efforts in providing it.

Thanks.

**Summary and General Comments**

Reviewer #1: This is an interesting statistical analysis and modelling of Giardia shedding and detection which could have implications on diagnostic tests assessments and estimations of disease burden.

Thanks for the positive comment.

Minor suggestions for language revisions:

288: …somewhat higher= …higher

We prefer to keep ‘somewhat’ to emphasize that differences are small.

291:  small differences = trivial differences

We’d prefer to keep our wording.

295:…somewhat lower= …lower

We again prefer to keep ‘somewhat’ to emphasize that differences are small.

390: rather inconsistently = inconsistently

Changed.

391: perhaps increased: increased

We’d prefer to keep ‘perhaps’, because (as Fig 4 shows) the differences are small and uncertain.

416: understand= understanding

We think our wording (“…critical to understand pathogen transmission…”) is correct here.

426: no support whatsoever= no support

Changed.

429: take imperfect test sensitivity = take the imperfect test sensitivity

We think our wording is correct here.

Reviewer #2: I think the paper makes a good contribution, and is really relevant to NTD community.  If possible, it would be good to simplify the presentation of the modeling and results, because it is hard for people not familiar with this approach to understand. Having a statistical reviewer familiar with these models would be good.

We tried our best to explain the ideas and methods as clearly as possibly, and are confident that most interested readers will be able to navigate the report without much difficulty.

Reviewer #3: The strength of this paper is the application of a hierarchical modeling approach to estimate Giardia infection in children attending kindergartens in the Federal District, Brazil while accounting (estimating) for stool-sample–level shedding rates and imperfect test-level detection. This same modelling approach could be used to separate lab-based diagnostics from disease related pathogen/parasite availability within field collected samples. The authors demonstrate this approach that could easily be used for other disease systems.

We thank the Reviewer for their positive assessment of the relevance of our work — also beyond the particular study system we used to illustrate the approach.

Reviewer #4: The manuscript presents an inspiring and insightful study on diagnostic tests and their limitations, using Giardia in children as a case study. It opens avenues for improving diagnostic sensitivity and provides a better understanding of the clinical and epidemiological characteristics of parasites. The study is well-designed, with information reported carefully, and while the work involves complex modeling with substantial calculations, the explanations are sufficient even for readers who may not be familiar with the subject. I commend the authors on their excellent work, and I hope this manuscript finds its deserved place in the scientific community. Here, I outline my points of confusion and suggestions for improvement. I would be happy to review a revised version and appreciate the authors' reasoning if they choose not to address certain suggestions.

Just as we did in our previous response, we thank the Reviewer for these very encouraging remarks.

Title:

The full title is important as it will represent the paper going forward. I believe it would be beneficial for the full title, like the short title, to specifically mention Giardia and modelling to better reflect the nature of the study. The current title doesn’t convey this adequately.

We appreciate and understand the Reviewer’s point. Our idea was to emphasize the generality of the approach. If the Reviewer and Editors agree, we would prefer to keep this more ‘general’ title. Otherwise, we would suggest adding to it a short, explicit mention to *Giardia*. For example,

“Disentangling the effects of intermittent faecal shedding and imperfect test sensitivity on the microscopy-based detection of gut parasites in stool samples: *Giardia* as a case-study”.

Abstract:

I think breaking down the sections of the abstract (Background, Methodology, Results, Conclusions) might help the reader gain a clearer understanding.

Done, thanks.

Author Summary:

If this section is aimed at a broader audience, particularly non-specialists, it would be useful to briefly explain the methodology, especially the hierarchical model used. Since the focus is more on the application of this model to estimate diagnostic success and its two key parameters, a short explanation of the model and its importance seems essential.

We really did our best to explain this quite technical report in 200 words (the upper limit as per the journal’s requirements). We have made a few small changes that may perhaps help — mainly using “statistical” instead of “hierarchical” in l. 56.

Introduction:

The introduction is well-written and structured, explaining fundamental issues in an accessible manner. However, the final paragraph could be written more clearly, as it is a bit confusing in how it lays out the two-level objectives of the study. Additionally, it might be beneficial to reference the background of the model used and how similar modeling approaches have previously been applied to other diseases, assisting researchers, clinicians, and policymakers.

To improve clarity and also follow the last suggestion by the Reviewer (see below), we have rephrased our objectives as a brief summary of the main study findings. We have also added refs. to key publications on the models and their application to infectious disease research (l. 123-130).

There are also somewhat repetitive explanations regarding the model on page 10, lines 242-247, page 13, lines 309-315, and page 18, lines 419-422, which echo what is stated in the final paragraph of the introduction. The authors may consider consolidating these explanations in one section and providing only summaries or omitting them in others.

We have removed the second repetitive bit (l. 325).

If this paper is examining something for the first time or has any other significant achievement, it would be helpful to briefly mention it at the end of the introduction.

As noted above, we have slightly rephrased the closing bits of the Introduction so that the goals of the study are presented as a brief summary of key study findings.

I hope these suggestions and comments prove helpful, and I look forward to seeing the revised manuscript.

We thank the Reviewer for the insightful points and useful suggestions, which have certainly improved our submission.
